# Individual placement and support (IPS): duration of employment support and equity of access and outcome in routine clinical practice

**DOI:** 10.1192/bjb.2024.68

**Published:** 2025-12

**Authors:** Miles Rinaldi, Rachel Perkins, Robert Baxter, Paul Dorrington, Kat Saville

**Affiliations:** 1 South West London and St George's Mental Health NHS Trust, London, UK; 2 Centre for Work and Mental Health, Nordlandssykehuset HF, Bodø, Norway; 3 Centre for Research and Education in Forensic Psychiatry, Haukeland University Hospital, Bergen, Norway; 4 ImROC, Nottinghamshire Healthcare NHS Foundation Trust, Nottingham, UK

**Keywords:** Evidence-based supported employment, individual placement and support, mental illness, vocational rehabilitation, implementation

## Abstract

**Aims and method:**

To explore the duration of support, reach, effectiveness and equity in access to and outcome of individual placement and support (IPS) in routine clinical practice. A retrospective analysis of routine cross-sectional administrative data was performed for people using the IPS service (*N* = 539).

**Results:**

A total of 46.2% gained or retained employment, or were supported in education. The median time to gaining employment was 132 days (4.3 months). Further, 84.7% did not require time-unlimited in-work support, and received in-work support for a median of 146 days (4.8 months). There was a significant overrepresentation of people from Black and minority ethnic communities accessing IPS, but no significant differences in outcomes by diagnosis, ethnicity, age or gender.

**Clinical implications:**

Most people using IPS services do not appear to need time-unlimited in-work support. Community teams with integrated IPS employment specialists can be optimistic when addressing people's recovery goals of gaining and retaining employment.

Work is central to the lives and well-being of most people, and has been an integral part of mental health services since the 1800s.^1^ People using mental health services often recognise that work provides meaning, purpose and promotes recovery, yet they experience low rates of employment and believe they are unlikely to gain employment.^2–6^

Individual placement and support (IPS) is a manualised form of supported employment, based on eight evidence-based principles:^7^ competitive employment is the goal, no exclusion criteria, attention to client preferences, rapid job search, job development based on client preferences, employment support and mental health treatment are integrated, welfare benefits counselling is provided and when in work there is time-unlimited support. Meta-analyses of IPS randomised controlled trials (RCTs) consistently find employment rates to be more than doubled for people with mental health conditions compared with other vocational rehabilitation approaches.^8^ IPS is recommended within the National Institute for Health and Care Excellence guidelines,^9,10^ and employment support is a quality standard for mental health services.^11^

However, questions remain about:

- Limited access to IPS resulting from low employment specialist case-loads and time-unlimited ongoing in-work support.^12^ Despite the absence of published UK data relating to duration of IPS support, anecdotal evidence suggests many people cease to need support after a relatively short period.
- The reach and effectiveness of IPS in routine clinical practice and, in particular, issues of equity in access to IPS, especially for those from Black and Asian minority ethnic communities.

## Method

### IPS service

South West London and St George's Mental Health NHS Trust provides mental health services across five London boroughs (Kingston, Merton, Richmond, Sutton and Wandsworth), serving a population of 1.1 million. The IPS service was developed with a 1:50 000 ratio of employment specialists to the general population, aligned with NHS England guidance.^13^ A total of 22 full-time equivalent (FTE) employment specialists are integrated members within the 18 multidisciplinary adult community mental health teams across the Trust, and are supervised by three lead employment specialists.

The employment specialists are part of community mental health teams, not a resource for the teams. They help teams identify people's recovery goals of gaining/retaining employment, receive referrals and then work closely with the individual and team to enable them to gain employment. Once settled in employment, and there is a shared decision that the support of an employment specialist is no longer needed, the person is discharged from the IPS case-load and support is provided by the clinical teams. The main tasks of the lead employment specialists include recruiting, training and providing active case-load management and outcome-based supervision to the employment specialists, modelling job development and liaising with the managers of the community mental health teams to maintain good integration of employment support and mental health treatment.

### Data and analysis

A retrospective analysis of routine cross-sectional anonymised administrative data was performed for all people who had used the IPS service between 1 April 2022 and 31 March 2023, with a follow-up period until 27 February 2024. This data included demographic characteristics (age, gender, ethnicity), most recent diagnosis, date of first contact with IPS, date of outcomes achieved and the date of discharge from the IPS case-load.

To explore the duration of IPS support received and to quantify two of the evidence-based IPS principles (rapid job search and time unlimited in-work support), the number of days (including weekends and holidays) was calculated from the date of first contact with IPS to gaining employment, and between gaining employment to discharge from the IPS case-load.

To establish whether there was equity in access and if the IPS case-load was representative of the adult community mental health teams case-load, a retrospective analysis of routine anonymised administrative data was performed for all people who had one or more contacts with these clinical teams between 1 April 2022 and 31 March 2023. The data contained demographic characteristics (age, gender, ethnicity) and diagnostic information. Although a default retirement age no longer exists in the UK, an upper age limit of 67 years was applied to reflect a working-age population who were likely to access IPS services. The ‘reach’ domain of the reach, effectiveness, adoption, implementation and maintenance (RE-AIM) implementation science framework^14^ was used to determine the IPS participation rate, and the representativeness between those who participated in IPS and the overall adult community teams case-load.

To understand whether there was equity in outcomes, the IPS Grow methodology for calculating employment rates was used.^15^ This calculates employment rates based on the number of unemployed people who gain employment. Those people who are already in work at their first IPS contact are not included in the calculation, and neither are those supported to complete their education goals. To ensure quality, and that IPS is delivered as intended, IPS fidelity was independently assessed by IPS Grow, using the IPS-25 scale.^16^ IPS Grow is the national implementation team who support the development, delivery and quality improvement of IPS, and are commissioned by NHS England and the Department for Work and Pensions to support the expansion of IPS across England (https://ipsgrow.org.uk). Quality is assessed via performance of IPS fidelity reviews, and quality marks are awarded to services based on this and outcomes achieved.

Data were compared between the IPS case-load and the adult community mental health teams case-load, and for those who gained employment versus those who remained unemployed. We tested for statistically significant differences by using chi-squared tests and *t*-tests to compare means, unless the data were highly skewed, where the Mann–Whitney *U* non-parametric test was used, and the median reported.

This was a service evaluation of the IPS service in South-West London. As such, formal ethical approval was not required.

## Results

Between 1 April 2022 and 31 March 2023, a total of 539 people used the IPS service (see Table 1). IPS fidelity was assessed four times (conducted at a borough level), with an average score of 103, indicating ‘good’ fidelity and achieved the IPS Grow charter quality mark. During this period, a total of 6165 people had one or more contacts with the adult community mental health teams, showing the IPS service reached 8.7% of the overall clinical case-load in a year.

**Table 1:** Demographic and diagnostic information of the individual placement and support case-load and the adult community mental health teams case-load, 1 April 2022 to 31 March 2023

		IPS case-load (*n* = 539)	Adult community teams case-load (*n* = 6165)	
Age (years)		Mean = 34.76 s.d. = 11.89 Range = 17–66	Mean = 41.44 s.d. = 13.54 Range = 17–67	*t* = 11.069, d.f. = 6702, *P* <0.001
Age bands	Up to 24 years	115 (21.3%)	798 (12.9%)	*χ^2^* = 105.164, d.f. = 4, *P* < 0.001
	25–34 years	179 (33.2%)	1419 (23.0%)	
	35–44 years	124 (23.0%)	1331 (21.6%)	
	45–54 years	81 (15.0%)	1271 (20.6%)	
	55–67 years	40 (7.4%)	1346 (21.8%)	
Ethnicity	White	282 (52.3%)	3759 (61.0%)	*χ^2^* = 18.835, d.f. = 4, *P* < 0.001
	Asian/Asian British	57 (10.6%)	596 (9.7%)	
	Black/Black British	112 (20.8%)	925 (15.0%)	
	Mixed ethnic group	26 (4.8%)	283 (4.6%)	
	Other ethnic group	62 (11.5%)	602 (9.8%)	
Gender	Male	249 (46.5%)	2807 (45.5%)	*χ^2^* = 0.188, d.f. = 1, *P* = 0.664
	Female	286 (53.5%)	3353 (54.5%)	
Diagnosis	Schizophrenia/psychosis	268 (49.9%)	2326 (50.3%)	*χ^2^* = 7.067, d.f. = 4, *P* = 0.132
	Bipolar disorder	68 (12.7%)	455 (9.8%)	
	Anxiety/depression	60 (11.2%)	598 (12.9%)	
	Personality disorder	73 (13.6%)	725 (15.7%)	
	Other non-psychotic	68 (12.7%)	524 (11.3%)	

IPS, individual placement and support.

### Rapid job search, time-unlimited in-work support and time on the IPS case-load

In quantifying the IPS evidence-based principles of rapid job search and time-unlimited in-work support, we found the median time from first contact with IPS to gaining employment was 132 days, or 4.3 months. Of those who gained employment, 84.7% (*n* = 177) did not require time-unlimited in-work support. They received in-work support for a median of 146.0 days, or 4.8 months.

Table 2 shows the distribution by time for those who gained employment. A total of 68.9% (*n* = 144) of people had gained employment by 6 months, 84.2% (*n* = 176) by 9 months and 93.8% (*n* = 196) by 12 months. Only 6.2% (*n* = 13) of people gained employment after 12 months. The findings are similar for time-unlimited in-work support. Of those who required in-work support, 61% (*n* = 108) required support for up to 6 months, a further 20.9% (*n* = 37) required support for up to 9 months, 10.2% (*n* = 18) required support for up to 12 months and only 8.0% (*n* = 14) required in-work support for more than 12 months.

**Table 2:** Time and type of support on individual placement and support case-load for those who gained employment and remained unemployed (*n* = 499)

Time on IPS case-load				
		Gained employment (*n* = 209)	Remained unemployed (*n* = 290)	
Time from referral to discharge on IPS case-load (days)		Median = 310.00 IQR = 199.0–420.5 Range = 20–1109	Median = 124.00 IQR = 64.0–195.0 Range = 5–566	*U* = 7838.00, *P* < 0.001
Discharged from IPS case-load		*n* = 177 (84.7%)	*n* = 267 (92.1%)	*χ^2^* = 6.745, d.f. = 1, *P* = 0.009
Remained on IPS case-load		*n* = 32 (15.3%)	*n* = 23 (7.9%)	
Total time on IPS case-load	0–3 months	*n* = 6 (2.9%)	*n* = 90 (31.0%)	*χ^2^* = 138.779, d.f. = 6, *P* < 0.001
	4–6 months	*n* = 31 (14.8%)	*n* = 103 (35.5%)	
	7–9 months	*n* = 33 (15.8%)	*n* = 37 (12.8%)	
	10–12 months	*n* = 41 (19.6%)	*n* = 21 (7.2%)	
	13–15 months	*n* = 31 (14.8%)	*n* = 12 (4.1%)	
	16–18 months	*n* = 19 (9.1%)	*n* = 2 (0.7%)	
	18 months or more	*n* = 48 (23.0%)	*n* = 25 (8.6%)	
Time to gaining employment and in-work support				
Time: IPS referral to job (*n* = 209)			Time: In-work ongoing support (*n* = 177)	
Median = 132 days (4.3 months) IQR = 77.5–222.2 s.d. = 112.99 Range = 11–527 days			Median = 146.0 days (4.8 months) IQR = 92.5–243.0 s.d. = 125.66 Range = 1–801 days	
0–3 months	*n* = 70 (33.5%)		0–3 months	*n =*43 (24.3%)
4–6 months	*n* = 74 (35.4%)		4–6 months	*n =* 65 (36.7%)
7–9 months	*n* = 32 (15.3%)		7–9 months	*n =* 37 (20.9%)
10–12 months	*n* = 20 (9.6%)		10–12 months	*n =* 18 (10.2%)
13–15 months	*n* = 7 (3.3%)		13–15 months	*n =* 7 (4.0%)
16–18 months	*n* = 6 (2.9%)		16–18 months	*n =* 4 (2.3%)
18 months or more	*n* = 0 (0%)		18 months or more	*n =* 3 (1.7%)

IPS, individual placement and support; IQR, interquartile range.

Table 2 shows there was a significant difference in the median time spent on the IPS case-load between those who gained employment and received time-unlimited in-work support (310 days or 10.2 months) compared with those who remained unemployed and were discharged from the IPS case-load (124 days or 4.1 months). It is interesting to note there was only a median of 8 days’ difference in employment support provided between those who gained employment and those who remained unemployed and were discharged from the IPS case-load.

### Equity in access

People using the IPS service were significantly younger, but representative with the overall case-load of the adult community mental health teams in terms of gender and diagnoses. Half of the IPS case-load (49.9%, *n* = 268) comprised people with a diagnosis of schizophrenia/psychosis. However, there was a significant difference in ethnicity: people from Black/Black British, Asian/Asian British, mixed and other ethnic groups were overrepresented on the IPS case-load, whereas White people were underrepresented. Table 1 shows the representativeness of the IPS case-load compared with the overall case-load of the adult community mental health teams.

### Equity in outcomes

A total of 93% (*n* = 499) of the IPS case-load consisted of unemployed people wanting to gain employment. Table 3 shows that 41.2% (*n* = 209) successfully gained employment. The mean age for those who gained employment was slightly younger than those who remained unemployed (33.5 *v.* 35.6 years) and was approaching statistical significance, but when aggregated into age bands, no significant difference was found; however, people aged 55 years and over did not achieve the same level of outcomes compared with younger people. There was no difference in employment outcomes by ethnicity: encouragingly, 47.8% of people from a mixed ethnic background, 44.2% from an Asian/Asian British background and 43.4% from a Black/Black British background gained employment. Similarly, there was no difference in outcomes by diagnosis, and it was positive to find that 49.2% of people diagnosed with a personality disorder and 44.8% of people diagnosed with schizophrenia/psychosis achieved their goals of gaining employment. There was no difference in outcomes between genders.

**Table 3:** Employment and unemployment outcomes with comparisons between demographic and diagnostic information (*n* = 499)

		Gained employment	Remained unemployed	
Employment outcomes		*n* = 209 (41.9%)	*n* = 290 (58.1%)	
Age (years)		Mean = 33.54 s.d. = 10.690 Range = 18–63	Mean = 35.63 s.d. = 12.725 Range = 18–66	*t* = 1.935, d.f. = 497, *P* = 0.054
Age	Up to 24 years	47 (44.3%)	59 (55.7%)	*χ^2^* = 5.773, d.f. = 4, *P* = 0.217
	25–34 years	74 (44.0%)	94 (56.0%)	
	35–44 years	48 (42.5%)	65 (57.5%)	
	45–54 years	31 (41.9%)	43 (58.1%)	
	55 years and over	9 (23.7%)	29 (76.3%)	
Ethnicity	White	105 (40.2%)	156 (59.8%)	*χ^2^* = 0.845, d.f. = 4, *P* = 0.934
	Asian/Asian British	23 (44.2%)	29 (55.8%)	
	Black/Black British	46 (43.4%)	60 (56.6%)	
	Mixed ethnic group	11 (47.8%)	12 (52.2%)	
	Other ethnic group	24 (42.1%)	33 (57.9%)	
Gender	Male	95 (40.9%)	137 (59.1%)	*χ^2^* = 0.039, d.f. = 1, *P* = 0.843
	Female	110 (41.8%)	153 (58.2%)	
Diagnosis	Schizophrenia/psychosis	112 (44.8%)	138 (55.2%)	*χ^2^* = 6.384, d.f. = 4, *P* = 0.172
	Bipolar disorder	26 (41.3%)	37 (58.7%)	
	Anxiety/depression	19 (33.3%)	38 (66.7%)	
	Personality disorder	32 (49.2%)	33 (50.8%)	
	Other non-psychotic	20 (32.3%)	42 (67.7%)	

Although the primary outcome of IPS is helping unemployed people to gain employment, there are other important outcomes: job retention (supporting people who are in work at their first IPS contact, to retain an existing job or find a new job) and supporting young people to complete their education. From the IPS case-load, 33 (6.1%) people were in work at their first IPS contact and were supported to retain their existing jobs. Their mean age was 37.2 years (s.d. = 10.732, range 20–59 years) and the majority were female (*n* = 21, 63.6%), of White ethnicity (*n* = 19, 57.6%) and were diagnosed with schizophrenia/psychosis (*n* = 11, 33.3%) or a personality disorder (*n* = 8, 24.2%). Additionally, seven people (1.3%) were also supported in mainstream education. Their mean age was 24.1 years (s.d. = 7.967, range 17–39 years) and the majority were male (*n* = 5, 71.4%), of Black/Black British (*n* = 3, 42.9%) or White ethnicity (*n* = 2, 28.6%), and all were diagnosed with a first episode of psychosis (*n* = 7, 100%).

## Discussion

This study reports on the duration of IPS support received and the reach, effectiveness and equity in access to, and outcomes from, IPS in routine clinical practice. The median time to gaining employment was 4.3 months, and most people did not require time-unlimited in-work support. They received in-work support for a median of 4.8 months. Those who remained unemployed spent a significantly shorter time on the IPS case-load. We also found that the IPS service reached almost 9% of the working-age population who are using adult community mental health teams in a year. Those who received IPS were younger, but representative compared with the adult community mental health teams case-load in terms of gender and diagnosis. Positive outcomes were achieved for a total of 46.2% of people who either gained or retained employment, or were supported to achieve their educational goals.

The IPS evidence-based principle of rapid job search can sometimes cause concern for mental health professionals, who may interpret this to mean people move rapidly into jobs; however, its actual meaning is that the job search process begins rapidly, within 30 days after first contact with IPS. This principle can also be an operational challenge – demand and capacity – as an IPS employment specialist's case-load is limited to a maximum of 20 clients in adherence to the highest possible score for IPS fidelity. Although the median time from first IPS contact to gaining employment was 132 days (4.3 months), by 3 months, 33.5% had gained employment, and by 9 months, most people (84.2%) had gained employment. Our findings are consistent with the first European IPS RCT, where employment outcomes were predominantly achieved within the first 9 months,^17^ and then replicated in the ‘IPS-Lite’ RCT in England that restricted job-seeking support to 9 months without significantly altering the effectiveness of IPS.^12^

Gaining and maintaining employment involves both social adaptation and psychological adjustment, and the evidence-based principle of time-unlimited in-work support is important to ensure people manage the transition into work and maintain employment.^18^ However, this principle also poses an operational challenge: how much support is needed and for how long? Although 84.7% of people who gained employment did not require time-unlimited in-work support, the majority of those (61%) required support for up to 6 months, with a further 20.9% requiring support for up to 9 months. Research suggests ongoing support promotes job tenure,^19^ but there are complex interacting individual, employment specialist and job-related factors that predict job tenure.^20^ Even so, our findings suggest people need longer periods of in-work support than found in the ‘IPS-Lite’ RCT, which limited in-work support to 4 months.^12^

Our findings align with the existing literature, in that the goal of gaining employment is both realistic and achievable for people using mental health services. Both the Royal College of Psychiatrists^21^ and the World Psychiatric Association^22^ recommend psychiatrists routinely explore a person's current employment status and consider appropriate work as a treatment outcome for any care provided as this can positively contribute to an individual's recovery. We found when IPS employment specialists are integrated into community mental health teams and teams addressed people's recovery goal of gaining employment, then 44.8% of people diagnosed with schizophrenia/psychosis and half of people (49.9%) diagnosed with a personality disorder gained employment. These findings are consistent with the IPS RCT literature showing people can gain and maintain employment regardless of diagnostic, clinical, functional and personal characteristics.^23^

Interestingly, this study found people from Black/Black British, Asian/Asian British, mixed and other ethnic group backgrounds were significantly overrepresented in accessing IPS, whereas people from White backgrounds were underrepresented. Although not significant, these findings followed through to employment outcomes, where 47.8% of people from mixed ethnic groups, 44.2% of Asian/Asian British people and 43.4% of Black/Black British people gained employment, whereas the lowest employment outcomes achieved, although very positive, were for people from a White ethnic background (40.2%). These findings are consistent with a previous study,^24^ and now provide evidence from across ten London boroughs that people from different ethnic groups appear not to be differentially disadvantaged in relation to either access to, or outcomes of, IPS services. These findings also align with the international IPS literature on ethnicity, and intersectional characteristics and outcomes.^25,26^

Although the primary outcome of IPS is helping people to gain employment, there are other important outcomes. Research shows people often come into contact with mental health services and are in work, but typically lose their jobs within a short period of time and do not return to work.^27,28^ Even after a short period of unemployment (3 months or more), people with mental health conditions are already less likely to return to work compared with people without mental health conditions, and they are particularly sensitive to the negative effects of unemployment and the loss of structure, purpose and identity.^29,18^ Additionally, enabling people to complete their education is a determinant of health, is associated with better employment prospects and people diagnosed with schizophrenia typically have poor educational outcomes.^30,31^ It is therefore important for IPS services to monitor and report these outcomes as part of service performance, recognising the clinically significant outcome of providing employment support to facilitate job retention or educational support to enable a person to complete their education and make the transition into the labour market.^32^

This study has several limitations. First, it is a naturalistic design and cannot attribute causality to the findings; however, we believe this limitation is also a strength, providing real-world evidence on the duration of IPS support needed and the effectiveness of IPS in routine clinical practice. Second, the findings for time-unlimited in-work support provide evidence that people who gained jobs were also maintaining them, but details of the types of jobs, hours worked, duration of employment and their experiences were unavailable. Although a previous evaluation found people worked in both a wide range of occupations and occupational levels,^33^ such details could have provided greater insight into whether people in this study were engaged in meaningful work. Third, employment specialist turnover affects service performance, productivity and the continuity of employment support provided within IPS services.^34,35^ During the study period, there was a level of employment specialist turnover, and we did not have access to the vacancy rate, which would have enabled us to reliably calculate the average employment specialist case-load size, and understand whether the reach of 8.7% of the overall clinical case-load was good or not. However, the reach found is greater than the 2% found in the USA.^36,^^37^

Since 2018, IPS services have been scaled up to be a core intervention within the transformation of community mental health services in England.^38^ The initial focus was to establish or expand existing IPS services to increase the capacity and quality (fidelity) of services and deliver against NHS England access targets. However, at an NHS Trust or Integrated Care Board level, questions arise in terms of the planning and performance of these services. While achieving equity in outcomes, the employment outcomes achieved in this study are consistent with the 44% outcomes found across European IPS RCTs,^39^ and suggest there is little to no research to practice gap. However, during the study period, the healthcare quality regulator, the Care Quality Commission,^40^ found only a quarter of people (26%) using mental health services in England reported they had ‘definitely’ received help and advice with finding work, and half (50%) did not receive help or advice but would have liked it. This suggests an unmet need for employment support for people using mental health services. Although there may not be an IPS research to practice gap, there might be a gap between those who want and need employment support, and those who have access to it.

## About the authors

**Miles Rinaldi** is an honorary senior research fellow at South West London and St George's Mental Health NHS Trust, London, UK; researcher at the Centre for Work and Mental Health, Nordlandssykehuset HF, Bodø, Norway; and researcher at the Centre for Research and Education in Forensic Psychiatry, Haukeland University Hospital, Bergen, Norway. **Rachel Perkins** is a clinical psychologist and senior consultant at ImROC (Implementing Recovery through Organisational Change) with the Nottinghamshire Healthcare NHS Foundation Trust, Nottingham, UK. **Robert Baxter** is a lead employment specialist at South West London and St George's Mental Health NHS Trust, London, UK. **Paul Dorrington** is a lead employment specialist at South West London and St George's Mental Health NHS Trust, London, UK. **Kat Saville** is a lead employment specialist at South West London and St George's Mental Health NHS Trust, London, UK.

## Data Availability

The data that support the findings of this study are available from the corresponding author, M.R., upon reasonable request.
